# Chemical Characterization and Biological Evaluation of *Epilobium parviflorum* Extracts in an In Vitro Model of Human Malignant Melanoma

**DOI:** 10.3390/plants12081590

**Published:** 2023-04-09

**Authors:** Sotiris Kyriakou, Venetia Tragkola, Ioannis Paraskevaidis, Mihalis Plioukas, Dimitrios T. Trafalis, Rodrigo Franco, Aglaia Pappa, Mihalis I. Panayiotidis

**Affiliations:** 1 Department of Cancer Genetics, Therapeutics & Ultrastructural Pathology, The Cyprus Institute of Neurology & Genetics, Nicosia 2371, Cyprus; 2 Department of Life & Health Sciences, School of Sciences & Engineering, University of Nicosia, Nicosia 2417, Cyprus; 3 Laboratory of Pharmacology, Medical School, National & Kapodistrian University of Athens, 11527 Athens, Greece; 4 Redox Biology Centre, University of Nebraska-Lincoln, Lincoln, NE 68583, USA; 5 School of Veterinary & Biomedical Sciences, University of Nebraska-Lincoln, Lincoln, NE 68583, USA; 6 Department of Molecular Biology & Genetics, Democritus University of Thrace, 68100 Alexandroupolis, Greece

**Keywords:** *Epilobium parvliforum*, UPLC-MS/MS, phytochemicals, cytotoxicity, anticancer, apoptosis, melanoma

## Abstract

Malignant melanoma is an aggressive type of skin cancer characterised by high metastatic capacity and mortality rate. On the other hand, *Epilobium parviflorum* is known for its medicinal properties, including its anticancer potency. In this context, we aimed to (i) isolate various extracts of *E. parviflorum*, (ii) characterize their phytochemical content, and (iii) determine their cytotoxic potential in an in vitro model of human malignant melanoma. To these ends, we utilized various spectrophotometric and chromatographic (UPLC-MS/MS) approaches to document the higher content of the methanolic extract in polyphenols, soluble sugars, proteins, condensed tannins, and chlorophylls -a and -b as opposed to those of dichloromethane and petroleum. In addition, the cytotoxicity profiling of all extracts was assessed through a colorimetric-based Alamar Blue assay in human malignant melanoma (A375 and COLO-679) as well as non-tumorigenic immortalized keratinocyte (HaCaT) cells. Overall, the methanolic extract was shown to exert significant cytotoxicity, in a time- and concentration-dependent manner, as opposed to the other extracts. The observed cytotoxicity was confined only to human malignant melanoma cells, whereas non-tumorigenic keratinocyte cells remained relatively unaffected. Finally, the expression levels of various apoptotic genes were assessed by qRT-PCR, indicating the activation of both intrinsic and extrinsic apoptotic cascades.

## 1. Introduction

The species of *Epilobium* comprise the largest genus of the plant family known as *Onagraceae*, with more than 165 species being reported worldwide [1]. The most common species include *Epilobium parviflorum* Schreb., *Epilobium hirsutum* L., *Epilobium rosmarinifolium* (*Epilobium dodonaei* Vill.), *Epilobium roseum* Schreb., and *Epilobium angustifolium* L. All these species are widely found across Europe and North America, and they are well known for their medicinal properties [2,3,4]. To this end, previous studies have documented the role of *Epilobium* extracts or infusions in the treatment of asthma, hiccups, and whooping cough [5,6]. In addition, other studies have documented the use of aqueous extracts and/or essential oils extracted from *E. parviflorum* for the treatment of diarrhoea, colitis, hormonal imbalance, as well as urinary and bowel-related diseases. In fact, these fractions have shown antimicrobial potency against a wide spectrum of bacteria and fungi, including *Enterococcus faecalis*, *Staphylococcus aureus*, *Escherichia coli*, *Pseudomonas aeruginosa* and *Candida albicans* [7,8,9]. On the other hand, recent studies have exploited extracts obtained from a variety of *Epilobium* species as potential anticancer agents. For instance, extracts of *E. angustifolium* L., *E. parviflorum* Schreb., and *E. hirsutum* L. were shown to induce apoptotic cell death in hormone-dependent prostate (LNCaP) and colon (HT-29) cancer cells, whereas others have shown a growth inhibition effect against human breast cancer (MCF-7) cells [10,11,12,13,14]. The phytochemical composition of various *Epilobium* species extracts revealed the presence of steroids (mainly *b*-sitosterol and its ester derivatives), triterpenes, macrocyclic tannins, fatty acids, and flavonoids (e.g., myricitrin, isomyricitrin, quercitrin, quercetin-3-*O*-*b*-*D*-glucuronide, and kaempferol) [2,15,16]. In particular, quercetin-3-*O*-glucuronide and myricetin-3-*O*-rhamnoside were shown to inhibit cyclooxygenases-1 and -2 (COX-1 and COX-2) [17]. In another study, oenothein B (an ellagitannin-based molecule) was shown to be capable of inhibiting myeloperoxidase, hyaluronidase, and lipoxygenase-5 (LOX-5) (in a plethora of *Epilobium* species) thereby inhibiting the formation of reactive oxygen species (ROS) [18,19,20,21].

The aim of the current study was to characterise the phytochemical content of various *E. parviflorum* extracts in an attempt to identify the fraction with the highest cytotoxic potency against an in vitro model of human malignant melanoma consisting of A375 and COLO-679 melanoma as well as non-tumorigenic immortalised keratinocyte (control; HaCaT) cells.

## 2. Results

### 2.1. Standardisation of UPLC/MS Conditions and Method Validation

For the determination of the most common polyphenols and flavonoids (including flavones and flavanols), selective ion recording (SIR) mode was used, whereas individual metabolites were quantified via multiple reaction monitor (MRM) transitions by using commercially available external standards. Extensive optimisation of the essential parameters regarding mobile phase, elution mode, flow rate, and stationary phase was performed to identify the optimum experimental condition(s) for the separation of the analytes and the generation of symmetrical signal peaks. For the determination of the optimum mobile phase, a combination of solvents (including methanol/water and acetonitrile/water) at different ratios was used; however, none of them contributed to the generation of symmetrical peaks. For this purpose, water was acidified with (0.1% *v*/*v*) formic acid, which facilitated the ionization of the analytes and also improved the peaks’ symmetry and shape. Concerning the stationary phase, the best separation was achieved on an ethylene bridged hybrid (BHE) column preheated at 40 °C. For the fragmentation of polyphenolic compounds, electrospray ionization (ESI) was employed in either negative or positive (ESI^±^) mode. The analytical method was validated according to the guidelines of the International Conference of Harmonization (ICH) [22]. Parameters including linearity, the limit of detection (LOD), the limit of quantification (LOQ), precision, and accuracy were determined for each of the examined analytes (Table 1).

**Figure 1:**
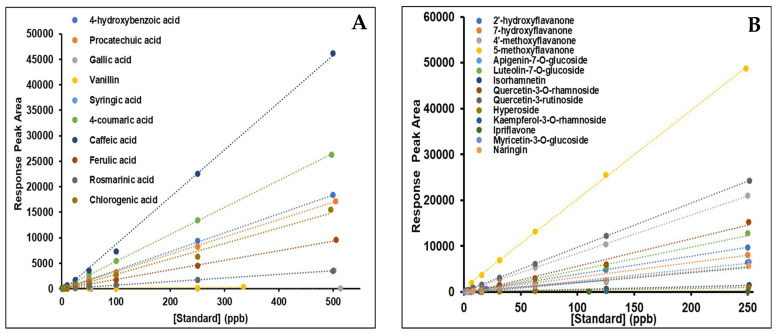
Calibration curves of the corresponding MRM fragments of (**A**) phenols; 4-hydroxybenzoic acid, protocatechuic acid, gallic acid, vanillin, syringic acid, 4-coumaric acid, caffeic acid, ferulic acid, rosmarinic acid and chlorogenic acid and (**B**) flavonoids; 2’-hydroxeflavanone, 7-hydroxyflavanone, 4-methoxyflavanone, 5-methoxyflavanone, apigenin-7-*O*-glucoside, luteolin-7-*O*-glucoside, isorhamnetin, quercetin-3-*O*-rutinoside, quercetin-3-*O*-rhamnoside, hyperoside, kaempferol-3-*O*-rhamnoside, ipriflavone, myricetin-3-*O*-glucoside and naringin at various concentrations (as shown in Table 1) used for the quantification of polyphenols in *Epilobium parviflorum* isolated extracts.

The standard curves generated from the standards were plotted with a linear regression equation of response peak areas as a function of various concentrations of compounds ranging from 0 to 500 ppb (Figure 1).

All screened compounds presented good linearity, whereas correlation coefficients (R^2^) were >0.99 for all analysed standards (Table 1). Finally, the reproducibility of the analytical methodology, utilising % of recovery, was evaluated (Table 1). For this purpose, each reconstituted extract of *E. parviflorum* was spiked with mixtures of standard solutions of polyphenolic compounds. Then, spike samples were prepared in triplicates, and the results were obtained from six repetitions. The % recovery was calculated according to Equation (1), where A was the final amount detected, A_0_ was the initial amount, and A*_a_* was the added amount:

(1) $$\%\;\mathrm{recovery}=\left(\frac{A-A_{0}}{A_{a}}\right)\times 100$$

The average recovery of all polyphenolic compounds ranged between 89.2% and 102.6%, thereby demonstrating the accuracy and reproducibility of the methodological approach.

### 2.2. Linearity, Accuracy, and Precision of the Methodology

The specificity and selectivity of the analytical protocol were evaluated using the limit of detection (LOD) and quantification (LOQ) values which were calculated based on the signal-to-noise ratio (S/N) pre-set at 3 and 10, respectively. The range of LOD and LOQ determination for polyphenolic compounds was 0.65–109.9 ppb and 1.21–105.2 ppb, respectively. Based on the values presented in Table 1, from all polyphenols, the LOD of *p*-coumaric acid was the lowest and that of gallic acid was the highest, suggesting that the sensitivity of *p*-coumaric acid was better than that of the other polyphenols ionised in ESI^−^. On the contrary, the highest sensitivity was shown to be for luteolin-7-*O*-glucoside and 7-hydroxyflavanone compared to the others ionised in ESI^+^. Finally, the degree of similarity of multiple samples within the same uniform was evaluated by utilising percent relative standard deviation (% RSD) in a way where six replicated samples (at the same concentration) were analysed within one to six continuous days for the estimation of intra- and inter-day precision, respectively.

### 2.3. Chemical Characterisation of the Isolated E. parviflorum Extracts

Among the three isolated extracts of *E parviflorum*, the methanolic one (MEEp) contained significantly higher concentrations of phenolic acids (1253.32 ± 19.12 μg of gallic acid eq/g of dry extract), followed by dichloromethane (201.12 ± 13.61 μg of gallic acid eq/g of dry extract) and petroleum (60.21 ± 2.25 μg of gallic acid eq/g of dry extract) (Table 2).

Particularly, ellagic acid (596.10 ± 1.44 μg/g of dry extract) was the most predominant one in MEEp, whereas caffeic (45.68 ± 1.14 μg/g of dry extract) and gallic (13.36 ± 1.53 μg/g of dry extract) acids were the most prevalent phenolics in dichloromethane and petroleum extracts, respectively (Table 2). In addition, a similar pattern was observed with flavonoids, as the highest content was found in MEEp (642.15 ± 10.35 μg of rutin eq/g of dry extract), followed by dichloromethane (311.68 ± 2.21 μg of rutin eq/g of dry extract) and petroleum (91.24 ± 1.11 μg of rutin eq/g of dry extract). The most abundant flavonoid in MEEp was apigenin-7-*O*-glucoside (64.10 ± 0.09 μg of rutin eq/g of dry extract), whereas in dichloromethane and petroleum extracts, the most abundant was 7-hydroxyflavanone (26.12 ± 1.10 μg of rutin eq/g of dry extract) and isorhamnetin (3.46 ± 0.13 μg of rutin eq/g of dry extract), respectively (Table 2). Determination of the pigment content revealed that MEEp contained elevated amounts of both chlorophyll-a (149.36 ± 7.23 μg/g of dry extract) and -b (254.98 ± 4.11μg/g of dry extract), followed by dichloromethane and petroleum. On the contrary, petroleum extract was enriched in lycopene (66.48 ± 0.27 μg/g of dry extract) and β-carotene (57.87 ± 0.01 μg/g of dry extract), compared to MEEp and dichloromethane (Table 2). In the case of total monoterpenoid content, the dichloromethane extract contained the highest concentration (52.23 ± 0.89 μg of linalool eq/g of dry extract) among the other two extracts. Moreover, MEEp contained the highest concentration of condensed tannins (224.21 ± 1.36 μg of catechin eq/g of dry extract), total water-soluble sugars (4.98 ± 0.56 nmols of mannose eq/g of dry extract), and proteins (287.36 ± 10.32 mg of BSA eq/g of dry extract), followed by dichloromethane and petroleum (Table 2).

### 2.4. Cytotoxic Profile of Various E. parviflorum Extracts

**Figure 2:**
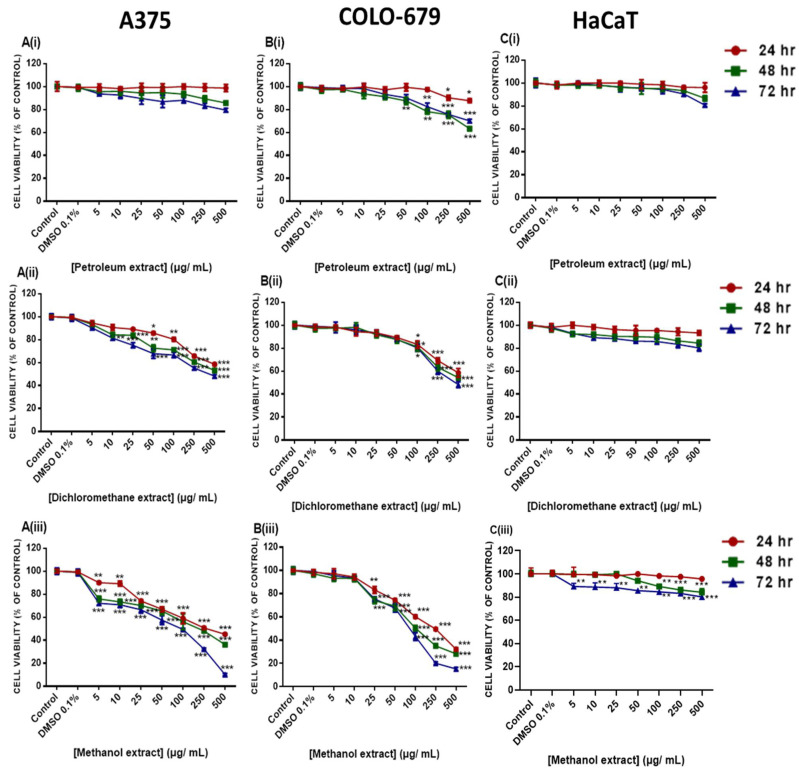
Cytotoxicity profiling of petroleum, dichloromethane, and methanolic *E. parviflorum* extracts in A375 (**A**(**i**–**iii**)) and COLO-679 (**B**(**i**–**iii**)) malignant melanoma cells, as well as non-tumorigenic keratinocyte (HaCaT) cells (**C**(**i**–**iii**)), exposed to 5–500 µg/mL of each extract, over 24–72 h. Data are expressed as means ± SEM and are representative of three independent experiments. Statistical significance is indicated at * *p* < 0.05, ** *p* < 0.01, and *** *p* < 0.001, relative to corresponding controls (0.1% *v*/*v* DMSO).

The cytotoxic potency of all three extracts was evaluated in human primary (A375) and metastatic (COLO-679) malignant melanoma cell lines using the Alamar blue assay (Figure 2A,B). A375 and COLO-679 cells were exposed to a range of concentrations (0–500 μg/mL) of each extract for 24–72 h. Results revealed that all three extracts exhibited a time- and concentration-dependent reduction in cell viability levels to a variable extent, as evidenced by their respective EC_50_ values (Table 3). Specifically, the petroleum extract demonstrated significant cytotoxicity only against the COLO-679 cell line at the higher concentrations used. However, no EC_50_ values were obtained (Figure 2A(i),B(i),C(i), Table 3). Moreover, the dichloromethane extract was shown to induce cytotoxicity at high concentrations in A375 (470.6 μg/mL) and COLO-679 (222.8 ± 7.51 μg/mL) cell lines but only at 72 h of exposure (Figure 2A(ii),B(ii),C(ii), Table 3). On the other hand, the MEEp exerted increased cytotoxicity in a concentration- and time-dependent manner. Specifically, a 50% reduction in cell viability levels was observed for both A375 (232.70 ± 2.98 at 24 h; 182.80 ± 4.19 at 48 h; and 63.30 ± 1.21 at 72 h) and COLO-679 (133.90 ± 3.24 at 24 h; 73.70 ± 1.21 at 48 h; and 68.90 ± 1.34 at 72 h) cells while non-tumorigenic keratinocyte (HaCaT) cells appeared relatively unaffected (Figure 2A(iii),B(iii),C(iii); Table 3).

Finally, COLO-679 cells were shown to be more sensitive to the cytotoxic effect of MEEp than A375 cells up to 48 h of exposure, an effect which was levelled out at 72 h. Consequently, our optimized experimental protocol included exposure(s) with MEEp, at an average concentration of 65 μg/mL, for both A375 and COLO-679 cell lines over a 72 h time period. On the other hand, the petroleum and dichloromethane extracts did not induce any cytotoxic effect on either of the cell lines. Finally, the HaCaT cells remained unaffected during the same experimental conditions. This is of utmost importance, as MEEp appears to induce cytotoxicity specifically against melanoma cells, whereas the adjacent neighbouring keratinocytes (represented by the HaCaT cells) are not affected.

### 2.5. Modulation of Apoptotic Gene Expression by Methanolic Extract of E. parviflorum

**Figure 3:**
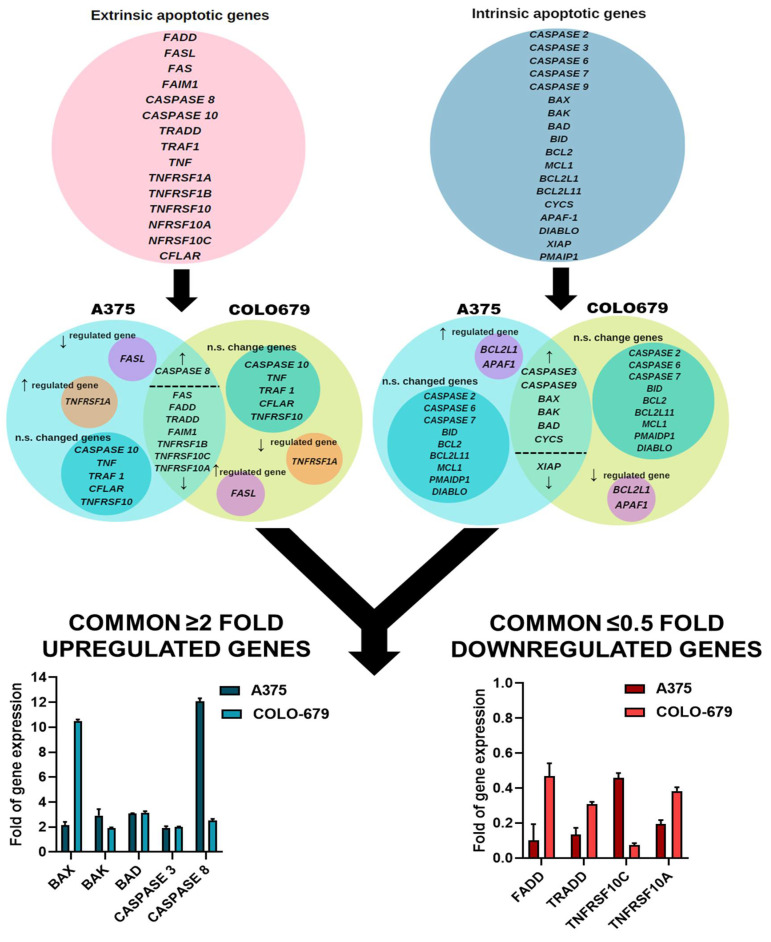
Panels of extrinsic and intrinsic apoptotic genes are shown in separate circles (red and blue, respectively). For each cell line, there are different circles indicative of genes being up- or down-regulated or remaining non-significantly (n.s.) changed. Light green panels indicate extrinsic or intrinsic apoptotic genes being common to both cell lines and either being up- or down-regulated. Finally, genes being up- or down-regulated by at least 2-fold or 0.5-fold, respectively (in either of the two cell lines), are also presented.

In this set of experiments, we have aimed to delineate the effect of MEEp in regulating apoptotic gene expression. For this reason, we utilized a panel of genes directly involved in apoptotic activation in A375 and COLO-679 cells. More specifically, the expression levels of specific genes involved in the extrinsic (e.g., *FADD*, *FASL*, *FAS*, *FAIM1*, *CASPASE-8*, *-10*, *TRADD*, *TRAF1*, *TNF*, *TNFRSF1A*, *TNFRSF1B*, *TNFRSF10*, *TNFRSF10A*, *TNFRSF10C*, and *CFLAR*), as well as intrinsic (e.g., CASPASES-3, -6, -7, -9, BAX, BAK, BAD, BID, BCL2, MCL1, BCL2L1, BCL2L11, CYCS, APAF-1, DIABLO, XIAP, and PMAIP1), apoptotic pathways were quantitated by qRT-PCR. Our results revealed that *FAS*, *FADD*, *TRADD*, *FAIM1*, *TNFRSF1B*, *TNFRSF10C*, and *TNFRSF10A* were down-regulated while expression of *CASPASE-8* was up-regulated in A375 and COLO-679 cells (Figure 3, Table 4).

Alternatively, expression levels of *CASPASE-10*, *TNF*, *TRAF1*, *CFLAR*, and *TNFRSF10* were not significantly changed in both cell lines. Moreover, the expression of *FASL* was specifically down-regulated in A375 while being up-regulated in COLO-679 cells, as opposed to *TNFRSF1A*, which was up-regulated in A375 but down-regulated in COLO-679 cells (Figure 3, Table 4). Furthermore, the analysis of the expression levels of intrinsic apoptotic genes revealed an up-regulation of *CASPASE-3* and *-9* as well as of *BAX*, *BAK*, *BAD*, and *CYS*, while only *XIAP* was down-regulated in both cell lines. The levels of *CASPASES-2*, *-6*, *-7*, *BID*, *BCL2*, *BCL2L11*, *MCL1*, *PMAIDP1*, and *DIABLO* remained unaffected in both cell lines. On the contrary, *BCL2L1* and *APAF1*, although up-regulated in A375, were down-regulated in COLO-679 (Figure 3, Table 4). Finally, we generated a panel of genes being either up-regulated by ≥2-fold or down-regulated by ≤0.5-fold in both cell lines. In this panel, *CASPASES-3* and -8, as well as *BAK*, *BAX*, and *BAD*, were up-regulated by ≥2-fold, whereas *FADD*, *TRADD*, *TNFRSF10A*, and *TNFRSF10C* were down-regulated by ≤0.5-fold, in both cell lines (Figure 3, Table 4). 

## 3. Discussion

It has been previously documented that malignant melanoma is characterized by its aggressiveness and increased metastatic capacity, and thus low survival rate. On the other hand, *Epilobium* is a plant with a variety of medicinal properties, including anti-proliferative activity, among others. In the present study, various fractions were obtained from the extraction of *Epilobium parviflorum* species by utilizing different solvents, such as petroleum, dichloromethane, and methanol. Overall, the differences in the polarity of each solvent reflect the differences observed in the compositional chemical analysis as well as the cytotoxicity profile. More specifically, the use of a non-polar solvent (e.g., petroleum) led to the isolation of significantly lower concentrations of polyphenols, sugars, proteins, condensed tannins, and monoterpenoids when compared to methanol. To this end, we documented that the methanolic fraction (MEEp) is enriched in flavonoids (apigenin-7-O-glucoside, isorhamnetin, myricetin-3-*O*-galactoside, kaempferol-3-O-rutinoside, and quercetin derivatives), phenols (gallic acid, protocatechuic acid, 4-hydroxybenzoic acid, ellagic acid, caffeic acid, and chlorogenic acid) and ellagitannins, with the latter being at considerably higher levels. Our data are in agreement with other studies reporting increased concentrations of polyphenols in *E. tetragonum*, *E. angustiofolium* L., *E. roseum*, and *E. montanum* L. species [23,24,25,26,27]. Such notable differences (in the levels of various phytochemicals) might be attributed to the different *Epilobium* species employed.

To the best of our knowledge, this is the first report demonstrating the cytotoxicity of *Epilobium* extracts in human malignant melanoma cells, although the findings of a previous report have shown the benefit of *E. angustifolium* L. as a potential herbal component of topical products for skin care and treatment [27]. Similar results have been supported by other studies exploiting the cytotoxic effects of *E. parviflorium* in other types of cancer, such as breast (MCF-7; EC_50_ = 73 μg/mL), prostate (PC-3; EC_50_ = 100 μg/mL and LNCaP; EC_50_ = 16 μg/mL), lung (A549; EC_50_ = 26 μg/mL), and colon (HT-29; EC_50_ = 73 μg/mL) carcinomas, as well as astrocytoma (1231N1; EC_50_ = 50 μg/mL) and neuroblastoma (SK-N-SK; EC_50_ = 50 μg/mL) [28,29,30]. Thus, the estimated EC_50_ value of 65μg/mL, as determined from our study, is comparable to the ones observed in all the above-mentioned studies. Moreover, it has been suggested that the effects of *Epilobium* species may be attributed to the presence of oenothein B (a strong antioxidant cyclic dimeric ellagitannin), which shows significant anti-proliferative, anti-neoplastic, and anti-migrative capacity against various cancers. This, in turn, might provide a rationale for a synergistic effect between oenothein B and other compounds within the mixture (e.g., polyphenols, pigments, sugars, and proteins), thereby contributing collectively to the marked anticancer activity of *Epilobium* species [31,32].

Our results suggest that MEEp can induce the activation of both the extrinsic and intrinsic apoptotic cascades by modulating the expression of several genes. To this end, the expression profile of a selective panel of intrinsic and extrinsic apoptotic genes demonstrated a (i) ≥2-fold up-regulation in the expression of *BAX*, *BAK*, and *BAD* in addition to *CASPASES -3* and *-8*, while it demonstrated a (ii) ≤0.5-fold down-regulation in the expression of *FADD*, *TRADD*, *TNFRSF10A*, and *TNFRSF10C* in both melanoma cell lines. Our data are in agreement with previous studies documenting depolarisation of the mitochondrial membrane and increased expression of pro-apoptotic proteins, by *E. parviflorum*, in human colorectal (HT-29 and BJ) cells [14]. In addition, another study has demonstrated that *Epilobium* extract induced the activation of caspase-3 (via mitochondrial depolarisation and consequent caspase-9 activation) in a model of human hormone-dependant prostate cancer (LNCaP) cells [11]. Finally, the findings of various studies have suggested that the mechanism of action of various *Epilobium* extracts (e.g., methanolic, ethanolic, and/or hydroethanolic) in inducing apoptosis is strongly related to their ability to induce mitochondrial membrane depolarisation and cytosolic release of cytochrome C, both of which can trigger the activation of caspase-9, thereby leading to the activation of caspase-3. In addition, the high abundance of macrocyclic ellagitannins (e.g., oenothein-B) in *Epilobium* extracts could be another factor contributing to their cytotoxicity by inducing elevated levels of ROS levels, which, in turn, can contribute to the activation of intrinsic apoptosis [33,34,35,36,37] via the mitogen-activated protein kinase and/or inositol triphosphate kinase pathways [38,39,40,41]. Finally, the induction of mitochondrial apoptosis through DNA damage cannot be excluded. To this end, ellagitannins (e.g., oenothein-B) can act as DNA binding agents capable of inducing DNA strand breakage [42]. Furthermore, the polar fractions of *Epilovium* (enriched in oenothein-B) were shown to inhibit DNA synthesis in an in vitro model of prostate carcinoma [43,44]. However, the exact mechanism(s) by which *Epilovium* species can induce cytotoxicity remain(s) largely speculative.

## 4. Materials and Methods

### 4.1. Materials

*Solvents:* chloroform, purity ≥ 99.8%, methanol LC-MS grade, purity ≥ 99.9%, water HPLC grade, acetonitrile HPLC grade, purity ≥ 99.9, and acetone ≥ 99.8%, were purchased from Honeywell (Medisell Nicosia, Cyprus); formic acid LC-MS grade was purchased from Thermofisher (Medisell, Nicosia, Cyprus); and sulfuric acid (95–97%), phenol, hydrochloric acid (37%), and *n*-butanol were purchased from Sigma-Aldrich (Saint Louis, MO, USA). *Analytical standards:* ascorbic acid and Trolox were purchased from Bioquochem (Asturias, Spain); catechin, mannose, and linalool and glutathione (GSH) were purchased from Sigma-Aldrich (Saint Louis, MO, USA); gallic acid, chlorogenic acid, ferulic acid, ellagic acid, vanillin, caffeic acid, syringic acid, *p*-coumaric acid, rosmarinic acid, 4-hydroxybenzoic acid, protocatechuic acid, 2′-hydroxyflavanone, 7-hydroxyflavanone, 4′-methoxyflavanone, 5-methyxyflavanone, apigenin-7-*O*-glucoside, luteolin-7-*O*-glucoside, isorhamnetin, quercetin-3-*O*-rhamnoside, hyperoside, myricetin-3-*O*-galactoside, kaempferol-3-*O*-rutinoside, ipriflavone, and naringin were purchased from Extrasynthese (Lyon, France); and bovine serum albumin (BSA) was purchased from Thermofisher (Medisell, Nicosia, Cyprus). *Reagents:* resazurin sodium salt and dimethyl sulfoxide (DMSO) were purchased from Sigma-Aldrich (Saint Louis, MO, USA); Macherey-Nagel NucleoZol RNA was purchased from Macherey-Nagel (Dueren, Germany); and PrimeScript^TM^ RT Reagent (Perfect Real Time) (PrimeScript^TM^ RT Reagent (Perfect Real Time), 5X PrimeScript Buffer (for Real-Time), PrimeScript RT Enzyme Mix I, Oligo dT Primer (50 μM), and Random 6-mers (100 μM) were purchased from TaKaRa, (Saint-Germain-en-Laye, France). *Assay kits:* Bicinchoninic acid (BCA) protein assay kit was purchased from Thermo Scientific (Waltham, MA, USA), and Total polyphenol assay kit was purchased from Bioquochem (Asturias, Spain). *Cell Culture reagents:* Dulbecco’s Modified Eagles Medium (DMEM) high glucose media, fetal bovine serum (FBS), *L*-glutamine, pen/strep (100 U/mL penicillin, 100 μg/mL streptomycin), trypsin-EDTA, and phosphate-buffered saline (PBS) were purchased from Biosera (Kansas City, MO, USA). All cell culture plastics were obtained from Corning (NY, USA).

### 4.2. Plant Material and Extract Preparation

Aerial parts of *Epilobium parviflorum* populations, consisting of leaves and flowers, were collected during blooming (June 2020) from the geographic region of northern Greece. The plant material was air-dried in the shade, packed in closed containers, and stored for phytochemical and biological studies. Briefly, the air-dried *E. parviflorum* plant parts were gradually extracted in a Soxhlet apparatus with petroleum ether, dichloromethane, and methanol. All extracts were concentrated to dryness under reduced pressure. A voucher specimen was kept in the herbarium of the Laboratory of Pharmacognosy, Department of Life & Health Sciences, School of Sciences & Engineering, University of Nicosia, under code *No 0619*-Eplbmpar, for future reference. Stock solutions of each of the *E. parviflorum* fractions were prepared in DMSO and kept at 4 °C, and protected from light until use. For the determination of the total phenolic (TPC), flavonoid (TPC), soluble sugar (TSSC), soluble protein (TSPC), and pigment contents, the dried fractions were solubilized in methanol or DMSO (1 mg/mL) and filtered through a 0.45 μm filter (mixed cellulose esters-Sartorius Stedim biotech, Guttenberg, Germany) and kept at −20 °C protected from light until used.

### 4.3. Determination of total Phenol, Flavonoid, Condensed Tannin, Monoterpenoid, Soluble Sugar, Protein, and Pigment Contents

Total phenol content (TPC) of all isolated fractions was evaluated by using a commercial polyphenol quantification assay kit (Bioquochem, Asturias, Spain) according to the manufacturer’s instructions. Determination of total flavonoid (TFC) and total soluble sugar (TSSC) contents was performed as previously reported with some modifications [45]. Determination of condensed tannin content was performed according to a previously published protocol [46]. Determination of total monoterpenoid content was performed by adapting a method previously reported [47]. Protein content was determined by utilizing the bicinchoninic acid (BCA) protein assay kit (Thermo Scientific, Waltham, MA, USA) according to the manufacturer’s protocol. The total content of chlorophylls-a and -b, lycopene, and β-carotene was determined as previously described [48]. Each content was calculated using the following Equations (2)–(5):

(2) $$\mathrm{Chlorophyll}-a\;(\mu g/g\;\mathrm{of}\;\mathrm{dry}\;\mathrm{extract})=\left[\frac{\left(0.999A_{663}-0.0989A_{645}\right)}{20}\right]$$

(3) $$\mathrm{Chlorophyll}-b\;(\mu g/g\;\mathrm{of}\;\mathrm{dry}\;\mathrm{extract})=\left[\frac{\left(1.77A_{663}-0.328A_{645}\right)}{20}\right]$$

(4) $$\mathrm{Lycopene}\;(\mu g/g\;\mathrm{of}\;\mathrm{dry}\;\mathrm{extract})=\left[\frac{\left(-0.0458A_{663}+0.204A_{645}+0.372A_{505}-0.0806A_{453}\right)}{20}\right]$$

(5) $$\beta -\mathrm{carotene}\;(\mu g/g\;\mathrm{of}\;\mathrm{dry}\;\mathrm{extract})=\left[\frac{\left(0.216A_{663}-1.224A_{645}-0.304A_{505}+0.452A_{453}\right)}{20}\right]$$

### 4.4. Preparation of Standards and Samples

Stock solutions of all analytical standards were prepared either in acetonitrile/water mixture (1:1) or in methanol/acetonitrile mixture (1:1) at a concentration of 1000 ppm. Working standard solutions were made by diluting each standard stock solution with ice-cold methanol. Each solution was kept in the dark and protected from light to minimize the autooxidation of polyphenols (mainly flavonoids) and pigments. In addition, stock standard and sample solutions were stored at −20 °C before use. All prepared solutions were passed through a membrane filter (0.22 μm, mixed cellulose esters) prior to UPLC-ESI-MS/MS analysis.

### 4.5. Liquid Chromatography Conditions

A Waters Acquity UPLC system (Waters Corp., Milford, MA, USA) was utilized. The chromatographic separation was performed on an ACQUITY UPLC BEH C18 (100 × 2.1 mm, particle size: 1.7 μm) column (Waters Corp., Milford, MA, USA) heated at 30 °C and eluted as previously reported with some modifications [49]. Briefly, the mobile phase consisted of a solution of acetonitrile (eluent A) and formic acid 0.1% (*v*/*v*) (eluent B). A flow rate of 0.3 mL/min was used, and the linear gradient conditions applied consisted of 5–100% A (0–4 min), 100–90% A (4.0–4.1 min), 90% A (4.1–5 min), 90–5% A (5–5.01 min), and 5% A (5.1–6 min). The injection volume was 10μL, and the autosampler temperature was set at 4 °C. For the MS/MS experiments, a Xevo Triple Quatrable (TQD) mass spectrometer detector (Waters Corp., Milford, MA, USA) was utilized in either positive or negative ionization mode (ESI±). Quantitative analysis was accomplished using selected multiple reaction monitoring (MRM) mode. The MRM conditions were optimized for each standard by MS manual tuning of each standard prior to sample analysis at a concentration of 1 ppm (Table S1, Figure S1). To acquire maximum signals, the optimized tuning parameters were set as follows: capillary voltage: 3.0 kV; cone voltage: 36 V; source temperature: 150 °C; desolvation temperature: 500 °C; source desolvating gas flow: 1000 L/h; and gas flow: 20 L/h. High-purity nitrogen gas was used as the drying and nebulising gas, whereas ultrahigh-purity argon was used as a collision gas. Data acquisition and processing were performed on MassLynx software (version 4.1).

### 4.6. Cell Culture

The human malignant melanoma cell lines (A375 and COLO-679) were purchased from the American Type Culture Collection (ATCC, Manassas, VA, USA) and Deutsche Sammlung von Microorganismen und Zellkulturen (DSMZ-Braunschweig, Germany), respectively. The human immortalized keratinocyte (HaCaT) cell line was kindly provided by Dr. Sharon Broby (Dermal Toxicology & Effects Group; Centre for Radiation, Chemical, and Environmental Hazards; Public Health England, Didcot, UK). A375 and HaCaT cells were cultured in DMEM (high glucose), whereas COLO-679 cells were grown in RPMI media. All media were supplemented with 10% fetal bovine serum (FBS), 2 mM L-glutamine, and 1% pen/strep (100 U/mL penicillin, 100 μg/mL streptomycin). Cells were cultured in a humidified incubator at 37 °C and 5%CO_2_, grown as monolayers, and sub-cultured at 80–90% confluency. All cell lines were cultured for 15–20 passages before new stocks were utilized.

### 4.7. Determination of Cell Viability

Determination of cell viability was performed by utilizing the Alamar Blue assay as previously described [46]. Briefly, cells were seeded in 100 μL/well into 96-well plates and incubated overnight before exposure with each *E. parviflorum* extract fraction. The density of A375 cells was 8000, 4000, and 2000 cells/well, whereas for HaCaT and COLO-679 cells, density was 10,000, 5000, and 2500 cells/well over 24 h, 48 h, and 72 h, respectively. On the following day, cells were exposed to a range of concentrations (e.g., 5–100 μg/mL) for each extract fraction (in 0.1% DMSO) over 24–72 h of exposure. For control conditions, cells were incubated with complete medium only or 0.1% *v*/*v* DMSO. At the indicated time points, 10 μL of resazurin dissolved in PBS (1 mg/mL final concentration) was added to each well and incubated for 4 h at 37 °C. The plates were then centrifuged, and the absorbance was recorded at 570 nm and 590 nm (reference wavelength) using a microplate reader (LT4500, Labtech, UK). Cell viability was expressed as a percentage of control cells’ viability.

### 4.8. Determination of Gene Expression

Total RNA was extracted from A375 and COLO-679 cells treated with 69 μg/mL of *E. parviflorum* methanolic extract, for 72 h, by utilising the Macherey-Nagel NucleoZol RNA isolation kit (Macherey-Nagel, Germany) and then stored at 4 °C overnight. Total RNA was measured using a NanoDrop™ One/OneC Microvolume UV-Vis Spectrophotometer (ThermoFisher Scientific, UK). For the reverse transcription, a PrimeScript^TM^ RT Reagent (Perfect Real Time) (TaKaRa, France) kit was used according to the manufacturer’s protocol. The samples were stored at −20 °C until further use. Real time-PCR was performed to validate the expression of various apoptotic genes. All reaction mixtures contained 1 μg of cDNA, SYBR green PCR master mix (Applied Biosystems, Waltham, MA, USA), and 10 μM of forward and reverse primers (Eurofins Genomics, Ebersberg, Germany) (Table S2), reaching a final reaction volume of 20 μL. Βeta-actin was used as a housekeeping gene for normalising data. PCR amplification took place utilizing the StepOne^TM^ Real-Time PCR System (Thermo Fisher Scientific, Waltham, MA, USA) for a total of 40 cycles. To verify specific amplification and the potential presence of primer dimers, a melting curve analysis was performed.

### 4.9. Statistical Analysis

Statistical comparison of all analyte contents was performed by one-way ANOVA coupled with a post hoc Tukey test for multiple comparisons. Statistical comparison between control and treated samples for cell viability levels, antioxidant, and relative gene expression levels was performed using a t-test by utilizing GraphPad Prism version 8.0.1 software.

## 5. Conclusions

In the current study, we characterized the chemical composition of various extracts (methanolic, dichloromethane, and petroleum) of *Epilobium parviflorum*. Our findings suggest that, among all isolated fractions, the methanolic one was enriched with a higher content of phenolic and flavonoid compounds together with condensed tannins, soluble proteins, and chlorophyll-b. On the other hand, it was shown that the methanolic extract also exhibited the highest anticancer potency against an in vitro model of malignant melanoma by means of reduced cell viability, when compared to dichloromethane and petroleum extracts. Moreover, we demonstrated that the increased cytotoxicity induced by the methanolic extract of *E. parviflorum* is, at least in part, due to the modulation of apoptotic gene expression, thereby conferring its potential anti-melanoma properties.

## Figures and Tables

**Table 1:** Limits of detection (LOD), quantification (LOQ), linearity, precision, and accuracy for all screened polyphenols. Calibration equations represent peak areas as a function of concentration (ppb). Intra- and inter-day experimental data were collected over a 6-day experiment, while % recovery data represent three independent experiments.

Compound	Linear Range (ppb)	LOD (ppb)	LOQ (ppb)	Calibration Equation ^a^	Correlation Coefficient (r^2^)	% RSD		% REC ^d^
						(Intra-day) ^b^	(Inter-day) ^c^	
POLYPHENOLIC COMPOUNDS								
4-hydroxybenzoic acid	3.01–499.50	3.01	14.20	y = 36.87x − 62.07	0.9991	1.15	2.21	98.8
Protocatechuic acid	0.66–504.50	0.66	14.70	y = 34.24x − 69.4	0.9995	1.25	2.65	86.3
Gallic acid	53.20–513.20	53.20	105.20	y = 0.67x − 1.5	0.9996	0.46	0.21	99.9
Vanillin	2.87–335.00	2.87	5.62	y = 0.67x − 0.1	0.9999	0.98	0.95	100.4
Syringic acid	2.01–501.60	2.01	2.86	y = 7.28x − 2.7	0.9996	1.36	1.01	96.6
p-coumaric acid	0.65–497.30	0.65	1.55	y = 52.84x + 36.9	0.9997	1.70	1.94	93.2
Caffeic acid	1.21–500	1.21	1.25	y = 92.95x − 344.4	0.9995	1.01	2.21	100.1
Ferulic acid	2.10–505.60	2.10	12.17	y = 19.02x − 68.4	0.9992	0.70	2.45	102.6
Rosmarinic acid	2.32–499.50	2.32	2.56	y = 7.03x + 12.34	0.9996	1.30	3.02	86.9
Chlorogenic acid	3.48–495.60	3.48	4.76	y = 25.02x + 60.3	0.9991	1.35	1.98	87.4
Ellagic acid	5.53–499.10	5.53	75.60	y = 2.18x + 7.4	0.9995	1.32	3.05	89.9
2′-hydroxyflavanone	19.50–250.00	19.50	20.12	y = 38.69x + 22.5	0.9998	2.70	4.32	99.5
7-hydroxyflavanone	1.97–249.90	1.97	2.21	y = 51.17x − 73.6	1.0000	2.63	1.42	98.9
4′-methoxyflavanone	2.21–250.00	2.21	3.89	y = 83.54x + 60.3	0.9999	2.89	1.87	93.6
5-methoxyflavanone	6.47–248.50	6.47	8.52	y = 195.14x − 493.9	0.9992	3.21	2.69	94.7
Apigenin-7-*O*-glucoside	1.87–125.30	1.87	4.42	y = 6.17x + 3.8	0.9998	3.48	2.54	95.8
Luteolin-7-*O*-glucoside	2.21–250.10	2.21	2.22	y = 51.52x − 89.9	0.9998	3.64	3.22	89.2
Isorhamnetin	14.01–251.1	14.01	2.31	y = 6.08x − 15.4	0.9992	2.48	1.18	100.1
Quercetin-3-*O*-rhamnoside	1.02–250.60	1.02	4.21	y = 60.83x − 38.6	0.9999	2.21	3.01	99.8
Quercetin-3-O-rutinoside	1.40–251.30	1.40	4.32	y = 97.74x + 109.7	0.9999	1.35	1.89	87.4
Hyperoside	6.32–249.90	6.32	3.21	y = 3.97x + 0.5	0.9998	2.14	1.37	96.3
Myricetin-3-galactoside	0.85–251.20	0.85	2.12	y = 26.38x − 31.8	0.9997	1.78	1.65	100.2
Kaempferol-3-*O*-rutinoside	0.76–250.00	0.76	1.21	y = 25.73x + 73.7	0.9997	1.36	2.21	91.2
Ipriflavone	109.90–250.00	109.90	13.21	y = 0.62x + 2.2	0.9994	1.69	1.11	93.6
Naringin	3.01–250.60	3.01	1.21	y = 22.88x − 43.3	0.9997	2.22	4.02	95.4

^a^ Chromatographic peak area (y) as a function of ppb concentration (x); ^b^ values are means of intra-day assays (*n* = 6); and ^c^ values are means of inter-day assays (*n* = 6); ^d^ (*n* = 3).

**Table 2:** Quantitative data demonstrating the phytochemical content of petroleum, dichloromethane, and methanolic extracts of *E. parviflorum*. N.D. indicates non-detectable metabolite levels; data are means of six independent experiments ± SD; all values with different superscript letters in a column are significantly different, while same superscript letters indicate no statistical significance, at *p* < 0.05.

	Various Extracts of *E. parviflorum*			
Phytochemical	Petroleum	Dichloromethane	Methanol	Expression Units
4-hydroxybenzoic acid	N.D.	18.20 ± 0.32 ^a^	18.40 ± 0.64 ^a^	μg/g of dry extract
Protocatechuic acid	1.92 ± 1.01 ^b^	20.42 ± 1.30 ^a^	21.95 ± 0.58 ^a^	
Gallic acid	13.36 ± 1.53 ^a^	38.93 ± 1.41 ^b^	101.25 ± 1.12 ^c^	
Vanillic acid	3.20 ± 0.01 ^a^	5.51 ± 0.002 ^b^	5.86 ± 1.00 ^c^	
Syringic acid	0.26 ± 0.001 ^a^	0.36 ± 0.01 ^a^	4.57 ± 0.69 ^b^	
*p*-coumaric acid	N.D.	12.30 ± 0.59 ^a^	15.28 ± 0.74 ^b^	
Caffeic acid	0.21 ± 0.001 ^a^	39.22 ± 1.21 ^b^	45.68 ± 1.14 ^c^	
Ferulic acid	N.D.	N.D.	4.48 ± 0.04	
Rosmarinic acid	N.D.	N.D.	6.31 ± 0.11	
Chlorogenic acid	N.D.	N.D.	7.24 ± 0.01	
Ellagic acid	N.D.	2.15 ± 0.10 ^a^	596.10 ± 1.44 ^b^	
Total phenols	60.21 ± 2.25 ^b^	201.12 ± 13.61 ^c^	**1253.32 ± 19.12 ^a^**	μg of gallic acid eq./g of dry extract
2′-hydroxyflavanone	N.D.	14.05 ± 0.02 ^b^	4.68 ± 0.17 ^a^	μg/g of dry extract
7-hydroxyflavanone	N.D.	26.12 ± 1.1 ^a^	34.59 ± 0.02 ^b^	
4′-methoxyflavanone	1.12 ± 0.01 ^a^	2.01 ± 0.02 ^b^	34.42 ± 0.03 ^c^	
5-methoxyflavanone	N.D.	N.D.	48.63 ± 0.03	
Apigenin-7-*O*-glucoside	N.D.	N.D.	64.10 ± 0.09	
Luteolin-7-*O*-glucoside	1.75 ± 0.68 ^a^	7.23 ± 0.21 ^b^	19.92 ± 1.00 ^c^	
Isorhamnetin	3.46 ± 0.13 ^a^	4.35 ± 0.15 ^b^	50.50 ± 0.13 ^c^	
Quercetin-3-*O*-rhamnoside	N.D.	0.95 ± 0.01 ^a^	1.65 ± 0.01 ^b^	
Quercetin-3-*O*-rutinoside	N.D.	N.D.	62.24 ± 0.02	
Hyperoside	N.D.	2.21 ± 0.01 ^a^	6.64 ± 0.02 ^b^	
Myricetin-3-galactoside	1.21 ± 0.63 ^a^	1.01 ± 0.001 ^a^	61.10 ± 0.12 ^b^	
Kaempferol-3-*O*-rutinoside	N.D.	1.11 ± 0.001 ^a^	32.30 ± 0.10 ^b^	
Ipriflavone	1.35 ± 0.03 ^a^	27.82 ± 0.12 ^b^	40.10 ± 0.67 ^c^	
Naringin	1.89 ± 0.11 ^a^	1.92 ± 0.01 ^a^	8.43 ± 0.06 ^b^	
Total flavonoids	91.24 ± 1.11 ^a^	311.68 ± 2.21 ^b^	**642.15 ± 10.35 ^c^**	μg of rutin eq./g of dry extract
Condensed tannins	41.78 ± 2.45 ^a^	59.54 ± 2.14 ^b^	**224.21 ± 1.36 ^c^**	μg of catechin eq./g of dry extract
Total monoterpenoids	10.21 ± 0.48 ^a^	**52.23 ± 0.89 ^c^**	21.11 ± 0.94 ^b^	μg of linalool eq./g of dry extract
Total soluble protein	7.48 ± 2.23 ^a^	49.65 ± 3.64 ^b^	**287.36 ± 10.32 ^c^**	mg of BSA eq./g of dry extract
Total soluble sugar	0.42 ± 0.02 ^a^	1.36 ± 0.02 ^b^	**4.98 ± 0.56 ^c^**	nmols of mannose eq./g of dry extract
Chlorophyll-a	36.89 ± 2.23 ^a^	49.54 ± 2.47 ^b^	**149.36 ± 7.23 ^c^**	μg of pigment/g of dry extract
Chlorophyll-b	10.26 ± 0.41 ^a^	39.14 ± 1.12 ^b^	**245.98 ± 4.11 ^c^**	
β-Carotene	**57.87 ± 0.01 ^b^**	4.45 ± 0.41 ^c^	0.24 ± 0.36 ^a^	
Lycopene	**66.48 ± 0.27 ^c^**	12.56 ± 1.56 ^b^	3.42 ± 1.48 ^a^	

**Table 3:** Determination of EC_50_ values (expressed as μg/mL) for all cell lines at each time point of exposure.

Time (hr)	Petroleum			Dichloromethane			Methanol		
	A375	COLO-679	HaCaT	A375	COLO-679	HaCaT	A375	COLO-679	HaCaT
	EC_50_ (μg/mL)								
24	N.D.	N.D.	N.D.	N.D.	N.D.	N.D.	232.7 ± 3.0	133.9 ± 3.2	N.D.
48	N.D.	N.D.	N.D.	N.D.	N.D.	N.D.	182.8 ± 4.2	73.7 ± 1.2	N.D.
72	N.D.	N.D.	N.D.	470.6 ± 5.7	222.8 ± 7.5	N.D.	63.3 ± 1.2	68.9 ± 1.3	N.D.

N.D. indicates non-detectable levels; IC_50_ values were calculated by utilizing the IC_50_ Tool Kit (http://www.ic50.tk/; accessed on 16 August 2022).

**Table 4:** Gene expression profiling of various intrinsic and extrinsic apoptotic genes in A375 and COLO-679 cell lines.

Genes	Cell Lines	
	A375	COLO-679
Intrinsic Apoptotic Pathway		
*CASPASE 3*	1.942 ± 0.124	1.996 ± 0.051
*BAX*	2.162 ± 0.262	10.510 ± 0.112
*BCL2L1*	4.506 ± 0.034	0.320 ± 0.243
*BCL2L11*	N.S.	N.S.
*CAPSASE 9*	1.732 ± 1.215	2.751 ± 0.427
*APAF-1*	1.565 ± 0.102	0.889 ± 0.076
*BAK*	2.920 ± 0.513	1.915 ± 0.063
*BID*	N.S.	N.S.
*CYCS*	3.259 ± 0.034	1.166 ± 0.139
*BAD*	3.087 ± 0.019	3.154 ± 0.114
*CASPASE 7*	N.S.	N.S.
*CASPASE 6*	N.S.	N.S.
*CASPASE 2*	N.S.	N.S.
*DIABLO*	N.S.	N.S.
*XIAP*	0.268 ± 0.072	0.828 ± 0.142
*BCL2*	N.S.	N.S.
*MCL1*	N.S.	N.S.
*PMAIP1*	N.S.	N.S.
**EXTRINSIC APOPTOTIC PATHWAY**		
*TNFRSF1A*	1.975 ± 0.491	0.618 ± 0.120
*FASL*	0.247 ± 0.646	1.107 ± 0.082
*CASPASE 8*	12.064 ± 0.257	2.531 ± 0.126
*FAS*	0.431 ± 0.050	0.610 ± 0.055
*CASPASE 10*	N.S.	N.S.
*TRADD*	0.135 ± 0.039	N.S.
*TRAF1*	N.S.	N.S.
*TNF*	N.S.	N.S.
*TNFRSF1B*	0.162 ± 0.134	0.684 ± 0.222
*FAIM1*	0.300 ± 0.121	0.552 ± 0.029
*FADD*	0.1020 ± 0.093	0.469 ± 0.073
*C-FLAR*	N.S.	N.S.
*TNFRSF10*	N.S.	N.S.
*TNFRSF10C*	0.459 ± 0.027	0.074 ± 0.120
*TNFRSF10A*	0.194 ± 0.232	0.384 ± 0.021

N.S. denotes non-significance.

## Data Availability

Not applicable.

## Supplementary Materials

The following supporting information can be downloaded at https://www.mdpi.com/article/10.3390/plants12081590/s1. Table S1. Multiple reaction monitoring conditions for polyphenolic compounds in UPLC-MS/MS analysis. Figure S1: UPLC- ESI-MS/MS chromatograms of polyphenolic compounds (including phenols and flavonoids) in various *Epilobium parviflorum* sample extracts. Table S2. Forward and reverse primers used for the determination of apoptotic gene levels.
