# Supplementary material for: Chemical Characterization and Biological Evaluation of Epilobium parviflorum Extracts in an In Vitro Model of Human Malignant Melanoma
Source: Plants (Basel). 2023 Apr 9;12(8):1590. doi: 10.3390/plants12081590 (PMC10146124; doi:10.3390/plants12081590)
Supplement: Supplementary file 1 [file plants-12-01590-s001.zip › plants-2263432-supplementary.pdf]

Chemical Characterization and Biological Evaluation of *Epilobium parviflorum* Extracts in an In Vitro Model of Human Malignant Melanoma

Sotiris Kyriakou [1], Venetia Tragkola [1], Ioannis Paraskevaidis [1], Mihalis Plioukas [2], Dimitrios T. Trafalis [3], Rodrigo Franco [4,5], Aglaia Pappa [6], and Mihalis I. Panayiotidis [1,*]

[1] Department of Cancer Genetics, Therapeutics & Ultrastructural Pathology, The Cyprus Institute of Neurology & Genetics, 2371 Nicosia, Cyprus
[2] Department of Life & Health Sciences, School of Sciences & Engineering, University of Nicosia, 2417 Nicosia, Cyprus
[3] Laboratory of Pharmacology, Medical School, National & Kapodistrian University of Athens, 11527 Athens, Greece
[4] Redox Biology Centre, University of Nebraska-Lincoln, Lincoln, NE 68583, USA
[5] School of Veterinary & Biomedical Sciences, University of Nebraska-Lincoln, Lincoln, NE 68583, USA
[6] Department of Molecular Biology & Genetics, Democritus University of Thrace, 68100 Alexandroupolis, Greece
[*] Correspondence: mihalisp@cing.ac.cy; Tel.: +357-22392626

Supplementary Material

Table S1. Multiple reaction monitoring conditions for polyphenolic compounds in UPLC-MS/MS analysis.

Polyphenolic Compound	Chemical Formula	Molecular Weight	$[M-H]^{\pm}$ (m/z)	MS^2 Fragments (m/z)	Cone Voltage (V)	Collision Energy (eV)	Retention Time (R_t)
4-hydroxybenzoic acid	$C_7H_6O_3$	138.12	136.95	65.00<br>93.00	23	25<br>13	1.88
Protocatechuic acid	$C_7H_6O_4$	154.12	152.95	108.95	25	13	1.64
Gallic acid	$C_7H_6O_5$	170.12	168.95	78.98<br>124.95	23	22<br>15	1.37
Vanillin	$C_8H_8O_3$	152.15	151.00	92.20<br>136.00	22	20<br>15	2.23
Syringic acid	$C_9H_{10}O_5$	198.17	197.00	122.95<br>182.00	27	23<br>13	1.93
p-coumaric acid	$C_9H_8O_3$	164.16	163.00	119.00	15	13	2.13
Caffeic acid	$C_9H_8O_4$	180.16	178.95	134.95	25	13	1.89
Ferulic acid	$C_{10}H_{10}O_4$	194.18	192.95	134.00<br>178.00	26	25<br>12	2.20
Rosmarinic acid	$C_{18}H_{16}O$	360.32	359.20	161.00<br>197.00	10	15<br>15	2.26
Chlorogenic acid	$C_{16}H_{18}O_9$	354.31	353.10	84.00<br>191.02	22	44<br>14	1.70
Ellagic acid	$C_{14}H_6O_8$	302.19	301.00	145.00<br>173.00	35	34<br>36	2.00
2′-hydroxyflavanone	$C_{15}H_{12}O_3$	240.27	239.00	119.30<br>93.10	40	25<br>16	3.42
7-hydroxyflavanone	$C_{15}H_{12}O_3$	240.27	239.05	135.20<br>91.15	41	25<br>23	3.42
4′-methoxyflavanone	$C_{16}H_{14}O_3$	254.29	255.15	240.00<br>161.30	31	17<br>22	3.78
5-methoxyflavanone	$C_{16}H_{14}O_3$	254.29	255.15	151.30	34	22	3.49
Apigenin-7-*O*-glucoside	$C_{21}H_{20}O_{10}$	432.38	431.15	268.35	35	22	2.15
Luteolin-7-*O*-glucoside	$C_{21}H_{20}O_{11}$	448.38	449.15	287.10	34	31	2.01
Isorhamnetin	$C_{16}H_{12}O_7$	316.28	315.00	151.00<br>300.20	43	30<br>20	2.86
Quercetin-3-*O*-rhamnoside	$C_{21}H_{20}O_{11}$	448.38	447.01	271.00<br>300.00	43	47<br>28	2.14
Quercetin-3-*O*-rutinoside	$C_{27}H_{30}O_{16}$	610.53	609.10	300.00<br>271.00	47	39<br>65	1.92
Hyperoside	$C_{21}H_{20}O_{12}$	464.38	463.30	300.00<br>271.15	47	24<br>44	1.99
Myricetin-3-galactoside	$C_{21}H_{20}O_{13}$	480.38	479.05	271.10<br>287.10	48	39<br>44	1.87
Kaempferol-3-*O*-rhamnoside	$C_{21}H_{20}O_{10}$	432.39	431.05	255.30<br>284.20	45	42<br>28	2.27
Ipriflavone	$C_{18}H_{16}O_3$	280.33	281.30	240.00	40	19	4.17
Naringin	$C_{22}H_{32}O_{14}$	580.54	579.15	271.10<br>151.50	45	33<br>40	2.21

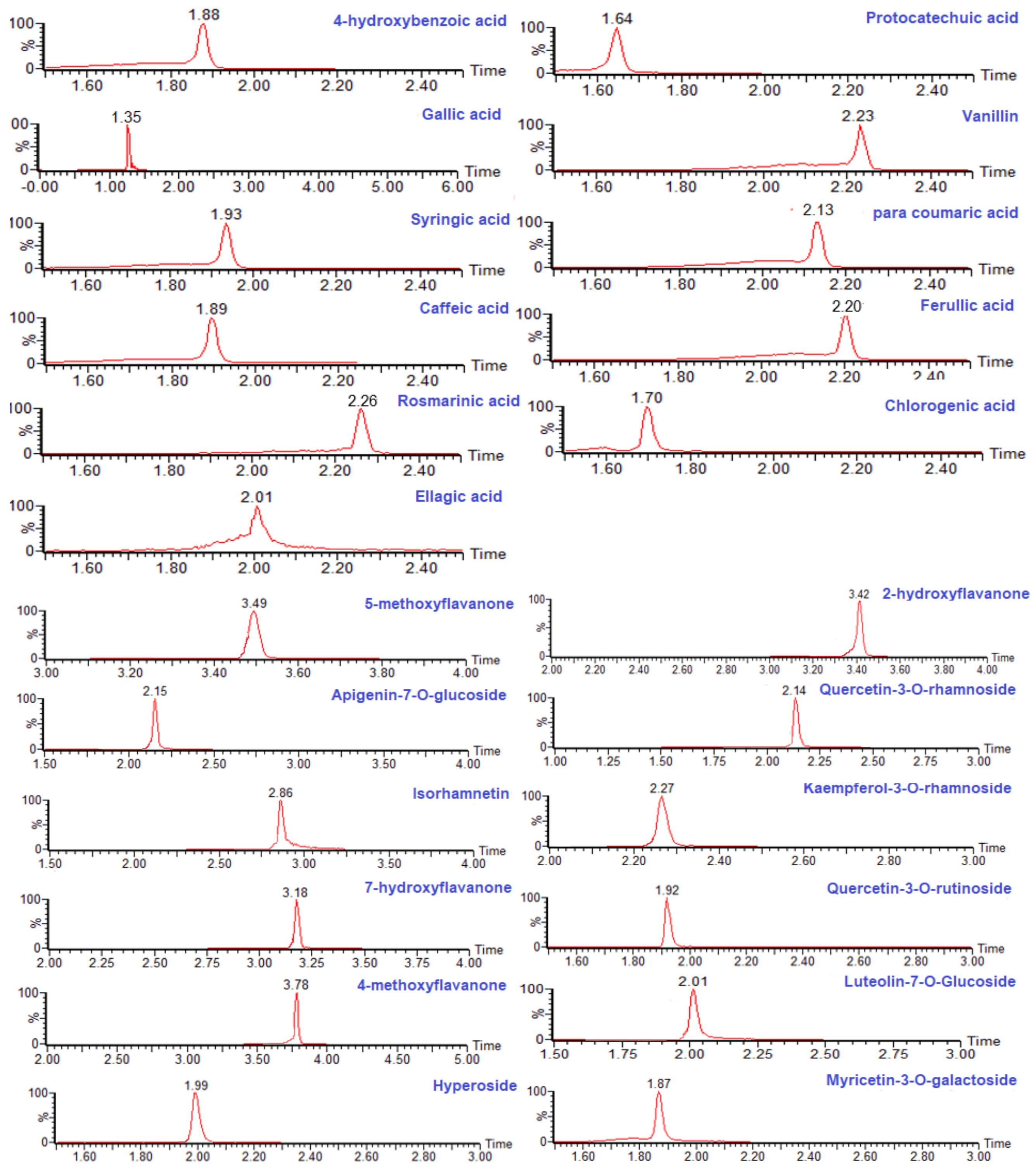

Figure S1. UPLC- ESI-MS/MS chromatograms of polyphenolic compounds (including phenols and flavonoids) in various *Epilobium parviflorum* sample extracts

Table S2. Forward and reverse primers used for the determination of apoptotic gene levels.

GENE	FORWARD PRIMER	REVERSE PRIMER
HUMAN *CASPASE 2*	ATTGGATCCCTTGGGCACCTC	ATCATGTCTGAGCGCGTGGG
HUMAN *CASPASE 3*	TTATTCAGGCCTGCCGTGGT	AGCATGGCACAAAGCGACTG
HUMAN *CASPASE 6*	TTTGGCTGCAATGAGCTCGG	GGCATCTGCGTGGCTAACAG
HUMAN *CASPASE 7*	AAGCTGAGGGAGCGTCCTAC	ACCGGTCTGGCTTAGCATCC
HUMAN *CASPASE 8*	GGCGGAGGGTCGATCATCTAT	TTCCTTCTCCCAGGATGACCC
HUMAN *CASPASE 9*	CGGTGACGCAAGAGCGAATC	GATCAGCTGCCTGGCCTGAT
HUMAN *CASPASE 10*	GGTAGTAATGAGGGCAGCTGTGT	TGAACCCAAGCCACTGGAACA
HUMAN *APAF1*	GTTGGGTTCATGGTGTGATG	TTTGTCTCCCAGAGCCTGA
HUMAN *BAD*	TCAGGGGCCTCGAGATCGG	TCCTGCTCACTCGGCTCAAA
HUMAN *BAX*	ACCTTGACTTGATTAGTGCCTTCT	GGGTCATCAATGAACTTGAGC
HUMAN *XIAP*	GCAAGAGCTAAGGAGACCA	AAGGGTATTAGGATGGGAGTTCA
HUMAN *FAS*	TTTTCCTCATGGCTTCACCT	ATGTGGCTGTGCTCATTGAC
HUMAN *BAK1*	AGACCTGAAAAATGGCTTC	CGGAAAACCTCCTCTGTGTC
HUMAN *BCL2*	AGTACCTGAACCGGCACCT	GCCGTACAGTTCCACAAAGG
HUMAN *BID*	TGCAGCTCAGGAACACCA	TCTCCATGTCTCTAGGGTAGGC
HUMAN *BCL2L11*	ACTGGAGAGCTCATTGCAGAC	AAATACCAGGACCCGAAGGT
HUMAN *BCL2L1*	CGTGGAAAGCGTAGACAAG	AAGAGTGAGCCCAGCAGAA
HUMAN *FASLG*	GAGTCTACCAGCCAGATGCACA	AGGCATGGACCTTGAGTTGG
HUMAN *TRADD*	TAGTGCAGCAGGAGGTGAGATG	CCAGCGAGGACTCCACAAAC
HUMAN *TRAF1*	TCATCAGGGCTTTCCTGCTCC	GTGGCCTCTGGGCTGTAAGAT
HUMAN *TNF*	TGCACTTTGGAGTGATCGGC	TTGTCACTCGGGGTTCGAGA
HUMAN *MCL1*	TTACGACGGGTTGGGGATGG	CTGCCCCAGTTTGTTACGCC
HUMAN *CYCS*	TTCGGAGCGGGAGTGTTCGT	TGTGGCACTGGGAACACTTCAT
HUMAN *DIABLO*	TGACTGCAGTTGGTCTTTCAG	GCGGTTATAGAGGCCTGATCT
HUMAN *CFLAR*	TCCATCTTGGGTGCGCCTTC	TCCGGGCCAGTCAACAGAAA
HUMAN *FADD*	CCGAGCTCAAGTTCTATGC	AGGTCTAGGCCGCTCTGC
HUMAN *FAIM1*	ATCGGAGCGAAGCAGAGAGG	CACAGTGGTTGAGCATGGGC
HUMAN *PMAIP1*	CAAGAACGCTCAACCGAGCC	AGGAGTCCCCTCATGCAAGT
HUMAN *TNFRSF1A*	TCCAAATGCCGAAAGGAA	CAATAATGCCGGTACTGGTTC
HUMAN *TNFRSF1B*	CCAGTGCGTTGGACAGAAGG	ATGGCCACCAGGGGAAGAAT
HUMAN *TNFRSF10*	TCACAGTGCTCCTGCAGTCT	GCCACTTTTGGAGTACTTGTCC
HUMAN *TNFRSF10A*	TACGCCCTGGAGTGACATCG	GACCCAAGCGCCAGAAACAC
HUMAN *TNFRSF10C*	CCCTAAAGTTCGTCGTCGTC	TGGTGGCAGAGTAAGCTAGGA
